# Closed-loop deep brain stimulation by pulsatile delayed feedback with increased gap between pulse phases

**DOI:** 10.1038/s41598-017-01067-x

**Published:** 2017-04-21

**Authors:** Oleksandr V. Popovych, Borys Lysyansky, Peter A. Tass

**Affiliations:** 1 Institute of Neuroscience and Medicine - Neuromodulation, Jülich Research Center, Jülich, Germany; 2 Department of Neurosurgery, Stanford University, Stanford, California, USA; 3 Department of Neuromodulation, University of Cologne, Cologne, Germany

## Abstract

Computationally it was shown that desynchronizing delayed feedback stimulation methods are effective closed-loop techniques for the control of synchronization in ensembles of interacting oscillators. We here computationally design stimulation signals for electrical stimulation of neuronal tissue that preserve the desynchronizing delayed feedback characteristics and comply with mandatory charge deposit-related safety requirements. For this, the amplitude of the high-frequency (HF) train of biphasic charge-balanced pulses used by the standard HF deep brain stimulation (DBS) is modulated by the smooth feedback signals. In this way we combine the desynchronizing delayed feedback approach with the HF DBS technique. We show that such a pulsatile delayed feedback stimulation can effectively and robustly desynchronize a network of model neurons comprising subthalamic nucleus and globus pallidus external and suggest this approach for desynchronizing closed-loop DBS. Intriguingly, an interphase gap introduced between the recharging phases of the charge-balanced biphasic pulses can significantly improve the stimulation-induced desynchronization and reduce the amount of the administered stimulation. In view of the recent experimental and clinical studies indicating a superiority of the closed-loop DBS to open-loop HF DBS, our results may contribute to a further development of effective stimulation methods for the treatment of neurological disorders characterized by abnormal neuronal synchronization.

## Introduction

Several neurological disorders like Parkinson’s disease (PD), essential tremor, epilepsy or tinnitus are characterized by abnormal neuronal synchronization^1–7^. High-frequency (HF) deep brain stimulation (DBS) is the gold standard for the treatment of medically refractory movement disorders and is currently being tested in other disease areas, too^8–11^. According to the standard stimulation protocol of HF DBS, a train of electrical pulses is administered at high frequencies (>100 Hz) to target areas like the thalamic ventralis intermedius (VIM) nucleus or the subthalamic nucleus (STN) via chronically implanted depth electrodes^8, 9^. The clinical and electrophysiological mechanisms of the symptom suppression by HF DBS is still an open issue^12–14^, and an increasing number of studies focus on the optimization of the therapeutic effects of HF DBS by appropriate calibration of the stimulation parameters and selection of appropriate stimulation targets^8, 12, 15–18^. One of the topics of these investigations is to evaluate the optimal shape and timing of the stimulation pulses^19–24^. This issue is also addressed in the present study and will turn out to be key for particularly effective closed-loop desynchronizing stimulation.

Another branch of research was devoted to a model-based development of novel stimulation algorithms specifically counteracting abnormal neuronal synchrony by desynchronization^25^. Initially the focus of these studies was on demand-controlled desynchronization stimulation^26–29^. To this end, specifically designed stimuli were delivered at demand-controlled times or periodically with demand-controlled stimulus strength^26–29^. Later on, stimulation-induced sustained desynchronization effects that persist cessation of stimulation came into focus^30^. This was because spike timing-dependent plasticity (STDP)^31, 32^ was taken into account in the model neural networks^30^. In computational studies coordinated reset (CR) stimulation^28, 29^, a spatio-temporally patterned desynchronizing stimulation technique, turned out to decrease the rate of coincidences and, in turn, to reduce abnormal synaptic weights^30, 33^. CR stimulation may cause an anti-kindling, i.e. an unlearning of both abnormal neural synchrony and synaptic connectivity by moving the neuronal population from a pathological attractor (with strong synaptic connectivity and neuronal synchrony) to a more physiological attractor (characterized by reduced synaptic connectivity and neuronal synchrony)^30, 33, 34^. As a result, CR stimulation may cause cumulative, long-lasting, sustained desynchronizing effects. Based on a number of computational studies dedicated to the application of CR stimulation with different stimulation modalities^28–30, 35–38^, several computational predictions were verified in pre-clinical as well as clinical studies. Sustained, long-lasting therapeutic and/or desynchronizing aftereffects induced by CR stimulation were revealed *in vitro* in rat hippocampal slices^39^, in monkeys rendered parkinsonian with the neurotoxin 1-methyl-4-phenyl-1,2,3,6-tetrahydropyridine (MPTP)^40, 41^, in human PD patients^42^ as well as in tinnitus patients^43–45^.

Other approaches suggested in the framework of the model-based development of desynchronizing methods are based on feedback techniques, where the mean field of synchronized population is measured, preprocessed and fed back as stimulation signal^46–56^, or on the phase response properties of neurons, where the stimulation signal can be derived from the phase response curve (PRC)^57, 58^. The feedback methods are intrinsically closed-loop techniques and posses a demand-controlled character, where the stimulation signal is significantly reduced or even vanishes as soon as desynchronization is achieved. The smooth and slowly oscillating stimulation signals of the feedback methods may however cause problems when directly administered to the neuronal tissue as electrical stimulation because of safety aspects like charge density limits^18, 59, 60^.

In this study we combine qualitatively different approaches to overcome their limitations and retain their upsides. We use HF trains of short charge-balanced stimulation pulses utilized for the standard HF DBS^16, 61^. The amplitude of the pulses is modulated by a slowly varying delayed feedback signal, by either linear delayed feedback (LDF)^46, 47^ or nonlinear delayed feedback (NDF)^50, 54^. We here show that this method constitutes *a pulsatile feedback stimulation* that fulfills safety requirements mandatory for electrical stimulation of neuronal tissue and inherits the desynchronization properties of the original delayed feedback techniques. We add a third ingredient by introducing an interphase gap between the cathodic and anodic phases of the single pulses of the HF pulse train. Intriguingly, a sufficiently large interphase gap turns out to significantly improve the desynchronizing outcome. In this study we apply the pulsatile linear as well as nonlinear delayed feedback with and without interphase gap to a physiology-based model network of STN-GPe neurons introduced previously^62, 63^ and investigate the resulting desynchronization effects.

As for standard HF DBS, each biphasic stimulation pulse is equipped with an interphase gap of finite width separating the first short pulse phase of large amplitude and the following recharging phase of longer duration and smaller amplitude^20, 61^. We focus on the effects induced by the interphase gap of different width. In a previous computational study it was shown that a properly chosen interphase gap may enhance action potential generation in silent neurons as well as entrainment of periodically bursting neurons^22^. In this study we show that the interphase gap can significantly improve the desynchronizing outcome of the pulsatile delayed feedback stimulation, so that a stronger desynchronization can be induced by a much smaller amount of the administered stimulation. We also demonstrate the robustness of the considered methods when the stimulation parameters vary.

Our novel approach may contribute to the paradigm of closed-loop DBS by employing safe stimulus trains adapted to the extent of abnormal neuronal synchrony and, in particular, achieving long-lasting effects by its specifically desynchronizing nature. In this way, our method might further improve the development of closed-loop DBS techniques which in first feasibility and proof of concept studies proved to be safe and showed promising results in comparison to standard open-loop DBS^64–72^.

## Methods

### Model

We consider a network of two neuronal populations, which models the dynamics of STN and GPe neurons. Each cell is modeled by the following system^62^:

1 $${C}_{m}v^{\prime} =-{I}_{{\rm{L}}}-{I}_{{\rm{K}}}-{I}_{{\rm{Na}}}-{I}_{{\rm{T}}}-{I}_{{\rm{Ca}}}-{I}_{{\rm{AHP}}}-{I}_{{\rm{syn}}}+{I}_{{\rm{app}}}+{I}_{{\rm{stim}}},$$

2 $$[{\rm{Ca}}]^{\prime} =\varepsilon (-{I}_{{\rm{Ca}}}-{I}_{{\rm{T}}}-{k}_{{\rm{Ca}}}[{\rm{Ca}}]),$$

3 $$X^{\prime} ={\varphi }_{X}({X}_{\infty }(v)-X)/{\tau }_{X}(v\mathrm{).}$$

In equations (1)–(3), *v* is a membrane potential of the neuron, the currents *I*_L_, *I*_K_, *I*_Na_, *I*_T_, *I*_Ca_, *I*_AHP_, *I*_syn_, and *I*_app_ are the corresponding leak, potassium, sodium, low threshold calcium, high threshold calcium, afterhyperpolarisation potassium, synaptic, and external current, respectively. [Ca] is the intracellular concentration of Ca^2+^ ions, and *X* = *n*, *h*, *r* are the gating variables.

The following currents from equation (1) attain the same form for both STN and GPe neurons:

$$\begin{array}{rllrll}{I}_{{\rm{L}}} & = & {g}_{{\rm{L}}}(v-{v}_{{\rm{L}}}), & {I}_{{\rm{K}}} & = & {g}_{{\rm{K}}}{n}^{4}(v-{v}_{{\rm{K}}}),\\ {I}_{{\rm{Na}}} & = & {g}_{{\rm{Na}}}{m}_{\infty }^{3}(v)h(v-{v}_{{\rm{Na}}}), & {I}_{{\rm{Ca}}} & = & {g}_{{\rm{Ca}}}{s}_{\infty }^{2}(v)(v-{v}_{{\rm{Ca}}}),\\ {I}_{{\rm{AHP}}} & = & {g}_{{\rm{AHP}}}(v-{v}_{{\rm{K}}})([{\rm{Ca}}]/([{\rm{Ca}}]+{{\rm{k}}}_{1})), &  &  & \end{array}$$

whereas current *I*_T_ is given by different expressions for the excitatory STN cells and for the inhibitory GPe cells:

$${\rm{STN}}:{I}_{{\rm{T}}}={g}_{{\rm{T}}}{a}_{\infty }^{3}(v){b}_{\infty }^{2}(r)(v-{v}_{{\rm{Ca}}}),\,{\rm{GPe}}:{I}_{{\rm{T}}}={g}_{{\rm{T}}}{a}_{\infty }^{3}(v)r(v-{v}_{{\rm{Ca}}}),$$

where ${b}_{\infty }(r\mathrm{)=1/(1}+\exp [(r-{\theta }_{b})/{\sigma }_{b}])-\mathrm{1/(1}+\exp [-{\theta }_{b}/{\sigma }_{b}])$. The functions *X*_∞_(*v*) and *τ*_*X*_(*v*) used in equation (3) and in the above definition of the currents read

$$\begin{array}{llll}{X}_{\infty }(v) & = & \mathrm{1/}(1+\exp [-(v-{\theta }_{X})/{\sigma }_{X}]), & X=n,h,r,m,s,a,\\ {\tau }_{X}(v) & = & {\tau }_{X}^{0}+{\tau }_{X}^{1}/(1+\exp [-(v-{\theta }_{X}^{\tau })/{\sigma }_{X}^{\tau }]), & X=n,h,r\mathrm{.}\end{array}$$

For GPe neurons *τ*_*r*_(*v*) = *τ*_*r*_ is a constant parameter.

**Figure 1:**
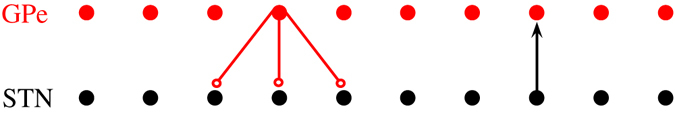
Coupling pattern of the STN-GPe neuronal network. Black circles depict STN cells, red circles depict GPe neurons. Each STN neuron excites a single GPe cell, whereas each GPe cell inhibits three STN neurons.

In our study we consider populations of *N* = 200 STN and 200 GPe neurons. The STN and GPe neuronal ensembles and coupling among them are schematically illustrated in Fig. 1. Each STN neuron excites a single GPe neuron, whereas each GPe neuron inhibits three neighboring STN neurons. We also consider periodic boundary conditions. The microscopic models of this type were introduced and investigated in a number of papers^62, 63, 73^, where STN neurons receive an inhibitory input from GPe neurons and, in turn, give an excitatory output to the GPe network.

The coupling among the neurons is realized via synaptic currents *I*_syn_ defined in the following way:

$${\rm{STN}}:{I}_{{\rm{syn}}}={g}_{{\rm{G}}\to S}(v-{v}_{{\rm{G}}\to {\rm{S}}})\sum {s}_{{\rm{j}}},\quad {\rm{GPe}}:{I}_{{\rm{syn}}}={g}_{{\rm{S}}\to G}(v-{v}_{{\rm{S}}\to {\rm{G}}})\sum {s}_{{\rm{j}}},$$

for STN and GPe cells, respectively. *j* is the index of neurons and summations are taken over all presynaptic neurons. The synaptic weights *g*_S→G_ = 0.4 nS/*μ*m^2^ and *g*_G→S_ = 1.7 nS/*μ*m^2^ reflect the strength of the coupling from STN neurons to GPe neurons, and in the opposite direction, respectively. The reversal potentials *v*_S→G_ = 0 mV and *v*_G→S_ = −100 mV reflect the excitatory coupling from STN to GPe neurons and inhibitory coupling from GPe to STN, respectively. The equation for the synaptic variables *s*_*j*_ reads:

4 $${s}_{j}^{^{\prime} }=\alpha {H}_{\infty }({v}_{j}-{\theta }_{g})(1-{s}_{j})-\beta {s}_{j},{H}_{\infty }(x)=\mathrm{1/}(1+\exp [-(x-{\theta }_{g}^{H})/{\sigma }_{g}^{H}])\mathrm{.}$$

We suppose that the neurons in the STN and GPe ensembles are nonidentical. For this, the applied currents *I*_*app*_ = *I*_app,*j*_ for STN cells are Gaussian distributed with the mean 10 pA/*μ*m^2^ and the standard deviation 0.015 pA/*μ*m^2^. The parameter *ε* = *ε*_*j*_ for GPe neurons are also Gaussian distributed with the mean 0.0055 ms^−1^ and the standard deviation 2 ⋅ 10^−5^ ms^−1^. The values of the other parameters for the STN and GPe neurons are listed in Supplementary Table S1.

### Synchronized dynamics

**Figure 2:**
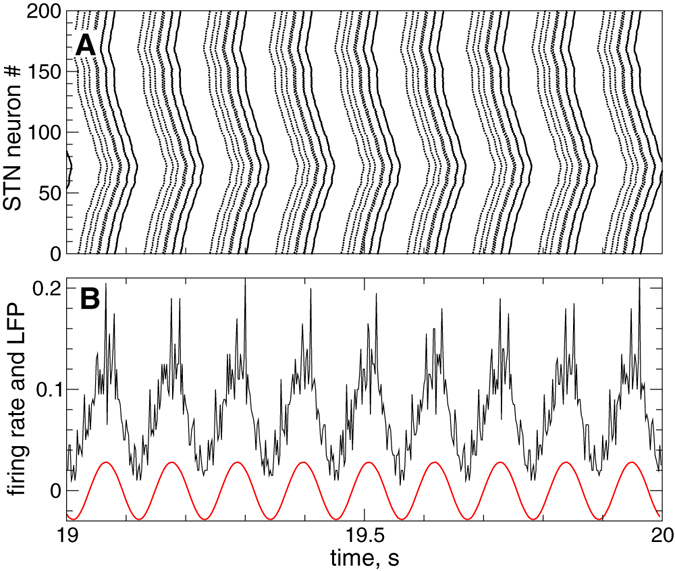
Synchronized dynamics of STN neurons (1) - (3) without stimulation. (**A**) Raster plot and (**B**) firing rate (relative number of neurons firing a spike at a given time) of STN neuronal activity. The red solid curve in plot (**B**) depicts the filtered LFP. The stimulation current *I*_*stim*_ = 0 pA/*μ*m^2^ in equation (1).

In Fig. 2 we illustrate the intrinsic dynamics of STN neurons interacting with GPe neurons. For the considered coupling between STN and GPe neurons, the STN neurons demonstrate a synchronized firing of bursts [Fig. 2A]. Such a synchronized neuronal dynamics results in a rhythmic activity of STN as reflected, for example, by the collective firing rate [Fig. 2B, black curve], which is the relative number of neurons firing a spike at a given time. We also calculate the STN local field potential (LFP) defined as $LFP(t)={N}^{-1}{\sum }_{j=1}^{N}{s}_{j}$, where *s*_*j*_(*t*) are the synaptic variables (4), and *N* is the number of STN neurons. The time course of the filtered LFP of synchronized STN neurons also demonstrates well-pronounced oscillations of larger amplitude [Fig. 2B, red solid curve], which can serve as an indicator of synchronized neuronal dynamics.

In this study we focus on the control of the collective synchronized dynamics of the STN-GPe network. The extent of synchronization can be estimated either by the LFP amplitude [Fig. 2B, red solid curve] or by the order parameter $R(t)=|{N}^{-1}{\sum }_{j=1}^{N}\exp (i{\psi }_{j}(t))|$ ^25, 74^, where *ψ*_*j*_(*t*) are the phases of individual neurons calculated from the neuronal bursting dynamics. The phase *ψ*_*j*_(*t*) of the *j*th neuron attains the values *ψ*_*j*_(*t*_*n*_) = 2*πn*, *n* = 0, 1, … at the time moments *t*_*n*_ of the burst onsets, i.e., the first spikes in the bursts, and linearly increases between the neighboring bursts *ψ*_*j*_(*t*) = 2*π*(*t* − *t*_*n*_)/(*t*_*n*+1_ − *t*_*n*_) + 2*πn* for *t* ∈ (*t*_*n*_, *t*_*n*+1_), *n* = 0, 1, …^75^. The order parameter *R*(*t*) ranges from 0 to 1 which correspond to the absence and presence of perfect in-phase synchronization, respectively. For example, the time-averaged order parameter 〈*R*(*t*)〉 ≈ 0.69 for the synchronized regime illustrated in Fig. 2.

### Feedback stimulation protocols

We investigate how the external stimulation based on delayed feedback can suppress the neuronal synchronization. We consider two different feedback stimulation techniques counteracting the neuronal synchronization. The first stimulation protocol is a linear delayed feedback (LDF)^46, 47^. To calculate the LDF stimulation signal, the LFP of synchronized STN neurons is measured and on-line filtered by applying a linear damped oscillator

5 $$\ddot{u}+{\alpha }_{d}\dot{u}+{\omega }^{2}u={k}_{{\rm{f}}}LFP(t).$$

Parameter *ω* approximates the frequency of the LFP oscillations *ω* = 2*π*/*T*, where *T* is the mean period of the LFP. For the synchronized state illustrated in Fig. 2, *T* ≈ 110 ms. As an output signal of equation (5), that is the filtered LFP [Fig. 2B, red curve], we use the variable $x(t)=\dot{u}$, which has a zero phase shift with respect to the original LFP signal^53^ and perfectly follows the oscillations of the firing rate, see Fig. 2B. The damping and scaling coefficients in equation (5) were chosen *α*_*d*_ = *k*_f_ = 0.008, which approximately preserves the amplitude of the input raw LFP signal.

The stimulation signal *S*(*t*) of the differential LDF is then calculated as^46, 47^:

6 $$S(t)=K(x(t-\tau )-x(t)),$$

where *K* is a dimensionless feedback gain and will be referred to as parameter of the stimulation intensity, and *τ* is the stimulation delay measured in milliseconds as the time in equations (1) - (3).

Another control method considered in this study is based on nonlinear delayed feedback (NDF) suggested in refs 50, 54, 76 for the control of pathological neuronal synchronization. To construct the stimulation signal, consider an analytic complex signal *Z*(*t*) = *x*(*t*) + i*y*(*t*), where the variable *x*(*t*) is the filtered LFP signal obtained with the help of equation (5) as for the case of LDF stimulation, and the corresponding *y*(*t*) signal can be calculated from *x*(*t*) by means of the Hilbert transform^75^. In a simple realization, which we use in this study, *y*(*t*) can be approximated by the time-shifted filtered LFP, *y*(*t*) = *x*(*t* − *T*/4), where *T* is the mean period of the LFP. The stimulation signal of the NDF is calculated as *S*_*z*_(*t*) = *KZ* ^2^(*t*)*Z* ^*^(*t* − *τ*), where the asterisk denotes the complex conjugacy. In our case we consider only the real part of *S*_*z*_(*t*) as the stimulation signal

7 $$S(t)=Kx(t-\tau )({x}^{2}(t)-{y}^{2}(t))+2Kx(t)y(t)y(t-\tau ),$$

where, as before, *K* is the stimulation intensity, and *τ* is the stimulation delay.

**Figure 3:**
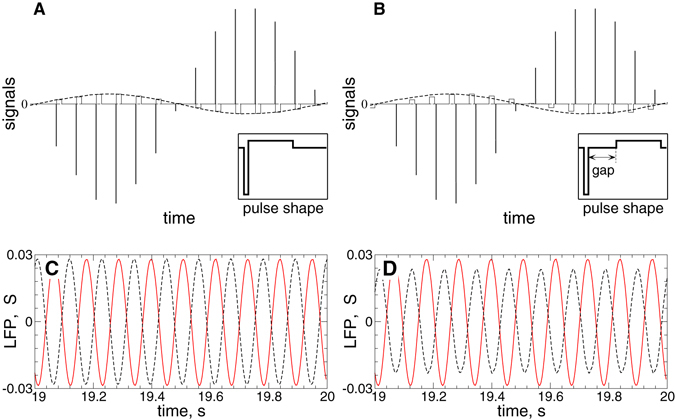
Stimulation signals of pulsatile delayed feedback. (**A**), (**B**) The amplitude of the high-frequency pulse train of charge-balanced asymmetric biphasic pulses (solid lines) is modulated by a slowly oscillating smooth signal *S*(*t*) of LDF (6) or NDF (7) depicted by dashed curves. The corresponding shape of a single pulse is schematically depicted in the inserts. In plot (**B**) the stimulation pulses contain an interphase gap between the cathodic and anodic phase of the pulse. (**C**), (**D**) Filtered LFP (red solid curves) from Fig. 1B and the corresponding feedback signals *S*(*t*) (black dashed curves) of (**C**) LDF and (**D**) NDF calculated from the LFP by means of equations (6) and (7), respectively. Stimulation delay *τ* = 50 ms, and the stimulation intensities (**C**) *K* = 0.5 and (**D**) *K* = 1000.

Since the stimulation signals (6) and (7) of the LDF and NDF, respectively, are derived from the filtered LFP [Fig. 2B, red curve], they are also smooth and slowly oscillating signals. Examples of the smooth feedback signals *S*(*t*) of LDF and NDF are illustrated in Figs. 3C,D by black dashed curves, respectively. Electrical stimulation of the brain with such signals, which may be referred to as smooth feedback stimulation, might cause an irreversible charge deposit in the vicinity of the electrode and lead to a damage of the neuronal tissue^18, 59, 60^. In order to satisfy safety requirements of electrical stimulation of neuronal tissue, we use a high-frequency pulse train of biphasic charge-balanced pulses utilized for standard HF DBS^16, 18, 61^. Each pulse consists of a cathodic and an anodic phase which deliver the same charge of opposite polarity providing, in such a way, a charge-balanced stimulation. This results in zero net charge injection into the stimulated tissue after each short biphasic pulse, and prevents from injury to nervous tissue^18, 59, 60, 77^. The amplitude of the pulses is modulated by the slowly oscillating feedback signal *S*(*t*) of LDF (6) or NDF (7) as schematically illustrated in Fig. 3A,B, where examples of the pulsatile stimulation current *I*_*stim*_ in equation (1) are shown. For a given smooth signal *S*(*t*) of the delayed feedback calculated according to equations (6) or (7) [Fig. 3, black dashed curves], the amplitude of a stimulation pulse is calculated at the time *t* = *t*_*p*_ of the pulse onset as *S*(*t*_*p*_). For the considered pulse shape [Fig. 3A,B, inserts] this value is assigned to the amplitude of the long, second phase of the pulse. The amplitude of the other, short counterpart of the pulse is obtained from the charge-balancing property such that the square delineated by the biphasic pulse is zero. We refer to the stimulation with such pulse trains modulated by the smooth LDF (6) and NDF (7) signals *S*(*t*) as *pulsatile LDF and NDF stimulation*, respectively.

The waveform of the biphasic charge-balanced stimulation pulses used for the standard HF DBS consists of a short first pulse (1st phase) of duration 60 to 450 *μ*sec^16^ followed by a longer charge-balancing 2nd phase of opposite polarity such that the total charge of the biphasic pulse is zero^61^, see the insert in Fig. 3A, where the considered case of the cathodic 1st phase is illustrated. Such a pulse shape is a standard pulse waveform widely used for HF DBS^61^. We use the standard frequency of 130 Hz for the HF DBS pulse train (the inter-pulse interval 1000/130 ≈ 7.69 ms)^16^. The width of the short pulse (1st phase) is taken as *PW* = 0.2 ms and relates to the duration of its long counterpart as 1:10, which is found to be energy efficient^21^.

In this paper we investigate the desynchronizing effect of the pulsatile delayed feedback stimulation for the cases when a small gap is introduced between the cathodic and anodic phases of the biphasic pulses as illustrated in the insert in Fig. 3B, see also refs 20, 22, 61. For the considered parameters of the pulse frequency and pulse length, the width of the interphase gap can range up to 5.49 ms, such that the neighboring pulses do not interfere with each other, see Fig. 3B, where the HF pulses with the gap width *GW* = 5 ms are modulated by a smooth feedback signal.

## Results

### Desynchronization by pulsatile feedback stimulation

We compare the effect of the pulsatile delayed feedback stimulation for different widths of the interphase gap, see Fig. 3B in Methods, when the stimulation is administered to synchronized STN neurons only (GPe neurons are not stimulated) whose stimulation-free dynamics is illustrated in Fig. 2 in Methods.

**Figure 4:**
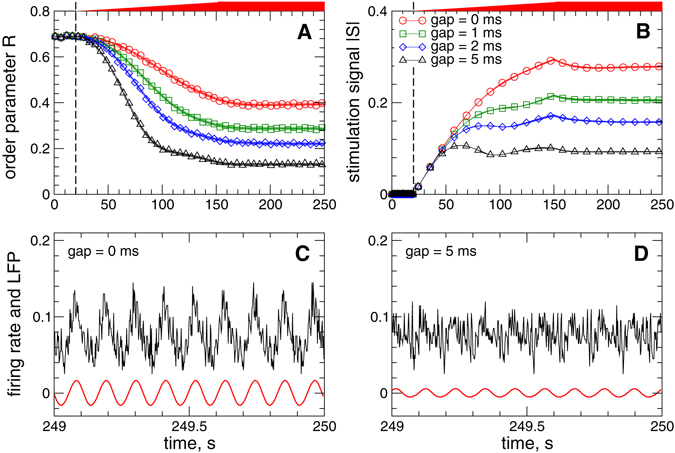
Suppression of synchronization in the neuronal ensemble (1) - (3) by pulsatile LDF stimulation (6). Time courses of (**A**) the order parameter *R* of STN neurons and (**B**) absolute value |*S*| of the feedback signal (6) are depicted for different widths of the interphase gap [Fig. 3B in Methods] as indicated in the legend in plot (**B**). Only local maxima of the oscillating signal |*S*| are shown in plot (**B**). The stimulation starts at *t* = 20 s indicated by vertical dashed lines, where the parameter of the stimulation intensity *K* linearly increases and reaches its maximal value *K* = 10 at *t* = 150 s as indicated by red bars on top of the plots. In plots (**C**) and (**D**) the corresponding time courses of the firing rate (black curves) and filtered LFP (red curves) are depicted for gap widths 0 ms and 5 ms as indicated in the plots. All simulations with stimulation delay *τ* = 70 ms.

Examples of the time courses of the order parameter *R*(*t*) of the STN neurons stimulated by the pulsatile LDF are illustrated in Fig. 4A for fixed parameters of the stimulation delay *τ* = 70 ms and stimulation intensity *K* = 10. After the onset of the stimulation at *t* = 20 s, the order parameter decays and saturates at a certain value smaller than that of the initial, pre-stimulation synchronized regime, which indicates a stimulation-induced desynchronization. Introducing the interphase gap of a finite width improves the desynchronizing impact of the pulsatile LDF stimulation, and a longer gap leads to a better desynchronization [Fig. 4A]. The large-amplitude oscillations of the firing rate and LFP of the stimulated STN neurons are much better suppressed by the pulsatile LDF stimulation with, for example, gap width *GW* = 5 ms [Fig. 4D] as compared to the case of zero gap [Fig. 4C]. The stronger desynchronization induced by the stimulation with larger interphase gap, but with the same stimulation intensity *K*, leads to a weaker stimulation necessary to reach such an extent of desynchronization, as illustrated in Fig. 4B.

**Figure 5:**
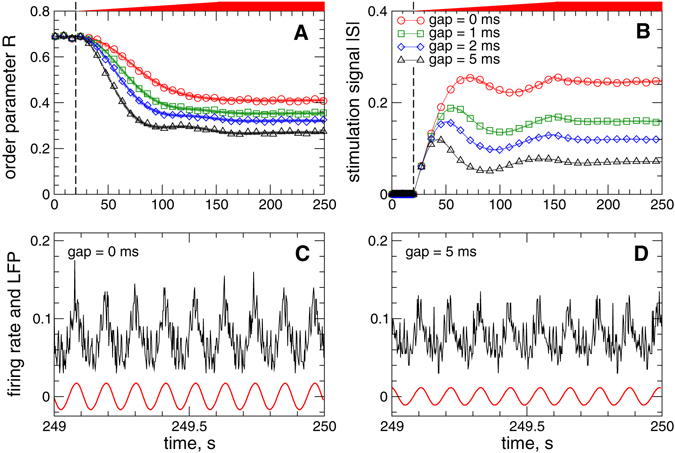
Suppression of synchronization in the neuronal ensemble (1) - (3) by pulsatile NDF stimulation (7). Time courses of (**A**) the order parameter *R* of STN neurons and (**B**) absolute value |*S*| of the feedback signal (7) are depicted for different widths of the interphase gap [Fig. 3B in Methods] as indicated in the legend in plot (**B**). Only the local maxima of the oscillating signal |*S*| are shown in plot (**B**). The stimulation starts at *t* = 20 s indicated by vertical dashed lines, where the parameter of the stimulation intensity *K* linearly increases and reaches its maximal value *K* = 50000 at *t* = 150 s as indicated by red bars on top of the plots. In plots (**C**,**D**) the corresponding time courses of the firing rate (black curves) and filtered LFP (red curves) are depicted for gap widths 0 ms and 5 ms as indicated in the plots. Parameter of the stimulation delay *τ* = 150 ms.

The same phenomenon of desynchronization enhancement by the interphase gap in the stimulation pulses is also observed for the pulsatile NDF stimulation, where the amplitude of the HF pulse train is modulated by the NDF signal (7), as illustrated in Fig. 5. Increase of the width of the pulse gap from *GW* = 0 ms to, for example, *GW* = 5 ms leads to a better desynchronization [Fig. 5A,C,D], whereas the amplitude of the stimulation signal is reduced by more than 3 times [Fig. 5B]. Therefore, the suggested modification of the stimulation pulses can significantly improve the desynchronizing effect of the pulsatile delayed feedback stimulation, where the stimulated neurons get better desynchronized by a much weaker stimulation. Below we evaluate the robustness and efficacy of the considered stimulation methods when parameters of the stimulation delay *τ* and the stimulation intensity *K* vary.

**Figure 6:**
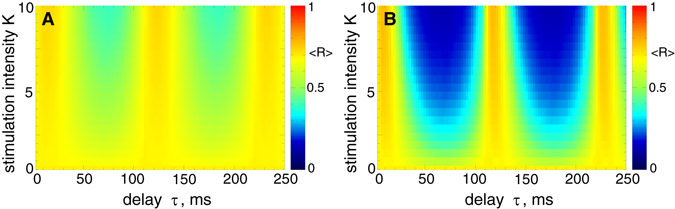
Impact of the pulsatile LDF stimulation (6) on the neuronal ensemble (1)–(3). The time-averaged order parameter 〈*R*(*t*)〉 of the stimulated STN neurons is depicted in color ranging from 0 (blue) to 1 (red) versus feedback delay *τ* and stimulation intensity *K* for the interphase gap width (**A**) *GW* = 0 ms and (**B**) *GW* = 5 ms.

The desynchronizing impact of the LDF stimulation can vary depending on the values of the stimulation parameters^46, 47, 54^. This property is illustrated in Fig. 6 for the pulsatile LDF, where the time-averaged order parameter 〈*R*〉 is depicted in color versus parameters of the stimulation delay *τ* and stimulation intensity *K*. For the stimulation with interphase gap of finite width, for example, *GW* = 5 ms [Fig. 6B], the parameter desynchronization regions become more pronounced, where the order parameter attains much smaller values as compared to the case of zero gap [Fig. 6A].

**Figure 7:**
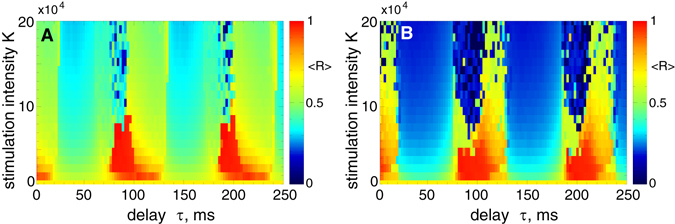
Impact of the pulsatile NDF stimulation (7) on the neuronal ensemble (1)-(3). The time-averaged order parameter 〈*R*(*t*)〉 of the stimulated STN neurons is depicted in color ranging from 0 (blue) to 1 (red) versus feedback delay *τ* and stimulation intensity *K* for the interphase gap width (**A**) *GW* = 0 ms and (**B**) *GW* = 5 ms.

The pulsatile NDF stimulation demonstrates a similar response to the variation of the stimulation parameters as pulsatile LDF stimulation for the considered model, as illustrated in Fig. 7. For the stimulation pulses with interphase gap [Fig. 7B], the neuronal synchronization can be suppressed much better by the pulsatile NDF stimulation than for the case of zero gap [Fig. 7A], as reflected by the values the order parameters 〈*R*〉. Moreover, the desynchronization regions occupy larger domains of the parameter space and increase in size for larger values of the stimulation intensity *K*. The favorable desynchronizing effect of NDF at large stimulation intensities has been revealed for several other models and stimulation setups^50, 54, 56, 76, 78^. We therefore investigate the behavior of the order parameter of the stimulated STN neurons while the parameter of the stimulation intensity *K* increases.

### Efficacy of pulsatile delayed feedback stimulation

**Figure 8:**
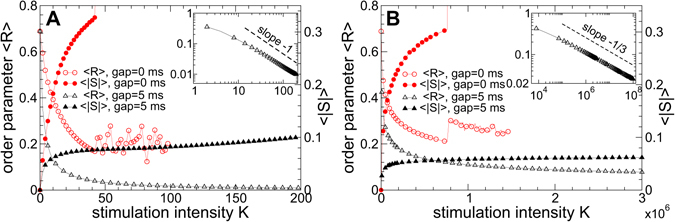
Synchronization control in the neuronal ensemble (1) - (3) by the pulsatile LDF and NDF stimulations for large stimulation intensity. The time-averaged order parameter 〈*R*〉 of the stimulated STN neurons and the time-averaged absolute value 〈|*S*|〉 of the feedback signal *S*(*t*) are depicted versus *K* for (**A**) pulsatile LDF stimulation (6) and (**B**) pulsatile NDF stimulation (7) for interphase gap widths *GW* = 0 ms and 5 ms as indicated in the legends. The scaling for 〈|*S*|〉 is given on the right vertical axes. In the inserts, 〈*R*〉 is plotted in the log-log scale for *GW* = 5 ms, where the dashed lines have the slopes (**A**) −1 and (**B**) −1/3 and are given for comparison. Parameter of the stimulation delay (**A**) *τ* = 70 ms and (**B**) *τ* = 150 ms.

For the pulsatile LDF stimulation we fix the optimal stimulation delay *τ* = 70 ms [Fig. 6] and continue the dynamics of the time-averaged order parameter 〈*R*〉 when the parameter of the stimulation intensity *K* slowly increases. We also calculate the corresponding time-averaged absolute value 〈|*S*|〉 of the smooth feedback signal *S* (6) which modulates the amplitude of the pulse train of the pulsatile LDF [Fig. 3 in Methods]. 〈|*S*|〉 can be considered as the amount of the administered stimulation. The results of the calculations are illustrated in Fig. 8A. As follows, the extent of desynchronization induced by the pulsatile LDF can be enhanced for larger values of the stimulation intensity, where the order parameter gradually decays as *K* grows. For the stimulation pulses without interphase gap, however, this process continues up to *K* ≈ 42, where the order parameter reaches 〈*R*〉 ≈ 0.17 [Fig. 8A, red empty circles] and then undergoes a jump toward larger values. Such a transition worsens the stimulation-induced desynchronization and leads to a much larger amount of the administered stimulation for larger stimulation intensity [Fig. 8A, red filled circles].

Introducing the interphase gap of a finite width to the stimulation pulses can improve the situation. For example, for *GW* = 5 ms, the order parameter 〈*R*〉 continues to decay also for relatively large values the stimulation intensity *K* [Fig. 8A, black empty triangles], and the pulsatile LDF stimulation with interphase gap can induce a well-pronounced desynchronization as reflected by small values of 〈*R*〉. Moreover, such a stimulation protocol requires a much smaller amount of the administered stimulation [Fig. 8A, black filled triangles], although it slowly increases as *K* grows. Such a behavior of the stimulation signal is connected to the decay rate of the order parameter 〈*R*〉 (and the amplitude of the LFP). We found that 〈*R*〉 decays according to a power law ~*K* ^*γ*^ with the exponent *γ* ≈ −1, as illustrated in the insert in Fig. 8A. For the considered network of *N* = 200 STN neurons, the direct numerical fit gives, however, *γ* ≈ −0.9, which may be caused by the finite-size effect^79^ and leads to a slow increase of the amount of administered stimulation as *K* grows.

The dynamics of the order parameter 〈*R*〉 and the amount of the administered stimulation 〈|*S*|〉 for the pulsatile NDF stimulation is illustrated in Fig. 8B versus parameter of the stimulation intensity *K* for selected optimal stimulation delay *τ* = 150 ms [Fig. 7]. As for the pulsatile LDF stimulation, the order parameter 〈*R*〉 gradually decays as *K* grows. When the stimulation pulses do not contain any interphase gap, the neuronal synchronization can be suppressed by the pulsatile NDF stimulation to a moderate level, and then the order parameters undergoes jumps to larger values [Fig. 8B, red empty circles]. This leads to a sudden increase of the amount of the administered stimulation for larger *K* [Fig. 8B, read filled circles]. If the stimulation pulses are equipped with an interphase gap, for example, with *GW* = 5 ms, the order parameter continues to decay to very small values, when *K* further increases [Fig. 8B, black empty triangles], and the stimulation-induced desynchronization is significantly improved.

In the insert in Fig. 8B the calculations are prolonged up to *K* = 10^8^, where the order parameter 〈*R*〉 is found to obey a power law ~*K* ^*γ*^ with *γ* ≈ −1/3. It is known that the NDF stimulation can suppress the amplitude of the mean field with the rate ~*K* ^−1/2^ as *K* increases if a single population of synchronized oscillators is considered^50, 54, 76^. For a network of two interacting populations, where only one population is measured and stimulated, the mean field of the stimulated neurons decays ~*K* ^−1/3^ as *K* grows^56, 78^. The latter case corresponds to the stimulation setup considered in this study, where only STN neurons from the STN-GPe network are recorded and stimulated. The performed numerical simulations of the considered network of *N* = 200 STN neurons result in the exponent *γ* ≈ −0.31 obtained by a numerical fit [Fig. 8B, insert], which may be caused by a finite-size effect^79^. Together with the nonlinear form of the feedback signal (7), this leads to a very slow increase of the amount of administered stimulation 〈|*S*|〉 as *K* grows [Fig. 8B, black filled triangles].

**Figure 9:**
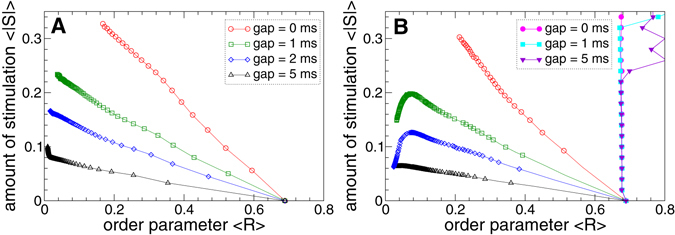
Efficacy of synchronization control in the neuronal ensemble (1) - (3) by the pulsatile LDF and NDF stimulations. The administered amount of the stimulation 〈|*S*|〉 is plotted versus the reached extent of desynchronization as given by values of the time-averaged order parameter 〈*R*〉 for (**A**) pulsatile LDF stimulation and (**B**) pulsatile NDF stimulation, where the amplitude of the stimulation pulses is modulated by delayed feedback signals (6) and (7), respectively. The width of the interphase gap is indicated in the legend in plot (**A**). Parameter of delay (**A**) *τ* = 70 ms and (**B**) *τ* = 150 ms. For comparison, in plot (**B**) the same quantities are depicted for the standard HF DBS (filled symbols) for the interphase gaps indicated in the legend. Note, desynchronization is achieved with standard HF DBS only at considerably greater amounts of stimulation, see Supplementary Fig. S2.

In order to estimate the efficacy of the pulsatile delayed feedback stimulation, we collect all data obtained by the variation of the stimulation intensity for different widths of the interphase gap and for fixed optimal stimulation delays, see Supplementary Section S2, and put the extent of the stimulation-induced desynchronization in relation to the amount of the administered stimulation. In this way, we plot the time-averaged absolute values 〈|*S*|〉 of the feedback signals (6) and (7) of the LDF and NDF stimulations, respectively, versus the corresponding values of the time-averaged order parameter 〈*R*〉 in Fig. 9. For the considered model, better desynchronization can be obtained for larger stimulation intensity *K* [Fig. 8] and can lead to a larger amount of the administered stimulation as discussed above. This can be seen in Fig. 9A for the pulsatile LDF stimulation, where smaller values of the order parameter 〈*R*〉 are always accompanied by larger values of 〈|*S*|〉.

For the pulsatile NDF stimulation the situation may however be different. For the stimulation pulses with interphase gap of intermediate width, the amount of the administered stimulation 〈|*S*|〉 can decay as the order parameter 〈*R*〉 decreases, in particular, for small 〈*R*〉 as illustrated in Fig. 9B. For such values of the order parameter which can be obtained for large stimulation intensity *K*, we found that the order parameter (and the amplitude of the LFP) can decrease faster than ~*K* ^−1/3^ [cf. Fig. 8B], i.e., with the exponent *γ* < −1/3, see Supplementary Section S2 for more detail. Due to the nonlinear form (7) of the feedback signal *S*, increasing stimulation intensity *K* can thus lead to a decay of both the order parameter and the amplitude of the stimulation signal, which can result in a better desynchronization obtained by a smaller amount of the administered stimulation [Fig. 9B] as has also been found for other models^50, 54, 56, 76, 78^. The same extent of desynchronization can thus require less stimulation for the NDF techniques as compared to the LDF stimulation method, if an interphase gap is introduced to the stimulation pulses, see Fig. 9A,B. For both considered pulsatile delayed feedback methods, the introduction of an interphase gap of a finite width significantly improves the efficacy of the desynchronizing stimulation as compared to the case of the stimulation pulses without gap. Moreover, longer interphase gap leads to a stronger desynchronization that can be induced by a much smaller amount of the administered stimulation [Fig. 9].

We compare the efficacy of pulsatile LDF and NDF in suppressing neuronal synchronization in the considered model to that of standard HF DBS. For this, we stimulate the synchronized STN neurons by the high-frequency pulse train of the considered charge-balanced pulses [Fig. 3] with constant amplitude, which corresponds to the constant modulating signal *S*(*t*) = *K* in Fig. 3. Such a pulsatile stimulation signal models that of the standard HF DBS. We found that strong HF DBS can destroy synchronization in the considered model, and introducing an interphase gap of a finite width may improve the stimulation outcome, see Supplementary Section S3 for details. However, the amount of the administered stimulation of HF DBS exceeds that of the pulsatile LDF and NDF by about an order of magnitude such that the delayed feedback methods significantly outperform HF DBS. For comparison, the amount of the stimulation administered by HF DBS, calculated as 〈|*S*|〉 = *K*, is plotted versus the order parameter of the stimulated neuronal population in Fig. 9B (filled symbols) for the range 〈|*S*|〉 ∈ [0, 0.35] of the plot, see also Supplementary Fig. S2B for larger scale of 〈|*S*|〉 = *K*. Obviously, pulsatile LDF and NDF are more efficient than HF DBS, where strong desynchronization can be obtained by pulsatile LDF and NDF by a much smaller amount of the administered stimulation than for HF DBS.

## Discussion

Closed-loop DBS is a stimulation paradigm for the treatment of medically refractory movement disorders that receives growing interest in the clinical arena^64–70, 72, 80^. The earlier theoretical developments of closed-loop control of abnormally synchronized neuronal dynamics suggested several methods based on either demand-controlled stimulation with specifically designed desynchronizing pulsatile stimuli^26–29^ or (delayed) feedback stimulation^46–56^. To realize the closed-loop stimulation, the activity of the controlled neurons has to be monitored permanently or intermittently, and stimulation is administered when necessary, or the stimulation strength is adapted to the extent of neuronal synchrony, or the stimulation signal is constructed directly from the measured activity^26–29, 46–56^. The population mean field (ensemble-averaged activity) reflects the collective synchronized dynamics of a population of interacting oscillatory neurons sufficiently well, and its large-amplitude oscillations are indicative of synchronization and can be used, e.g., to trigger stimulation onset.

Based on the above idea, a proof of principle of a closed-loop adaptive DBS (aDBS) in PD patients was reported in ref. 66, where the onsets and offsets of HF DBS were triggered by threshold crossings by LFP assessing beta-band STN activity with delay 30 to 40 ms for the stimulation onset with respect to the threshold crossing by LFP. The average improvement in clinical motor scores in the aDBS condition was significantly better by about 30% despite delivering less than 50% of the stimulation current as compared to the conventional continuous HF DBS (cDBS) condition^66^. In addition, aDBS employing real-time feedback from the ongoing LFP oscillations was more effective than random intermittent DBS^66^.

So far, in several studies the strength of stimulation was adapted to the extent of abnormal synchrony more or less continuously in time in a closed-loop setting. In a computational study with demand-controlled desynchronizing CR stimulation the strength of the periodically delivered pulsatile stimuli (more precisely, the length of the HF pulse trains used for CR stimulation) was gradually adapted to the amount of synchrony^28^. In a single-case study in a Parkinson’s patient the stimulation voltage was linearly updated each second based on the LFP beta band power^72^. An extreme variant of continuous adaptation of the stimulation strength to the extent of abnormal neuronal synchrony is realized by the standard (i.e. smooth) delayed feedback stimulation techniques, linear delayed feedback^46, 47^ and non-linear delayed feedback^50, 54, 56, 76, 78^. In the present study we consider a similar approach, where the amplitude of the HF DBS pulse train is modulated by a smooth signal [Fig. 3]. On the one hand, the employed HF DBS pulse train of charge-balanced pulses satisfies safety requirements mandatory for electrical stimulation of neuronal tissue. On the other hand, the modulation signal is calculated according to the algorithms of LDF^46, 47^ or NDF^50, 54, 56, 76, 78^, which have been suggested for effective desynchronization of abnormally synchronized neuronal populations by demand-controlled stimulation before. The combined stimulation signal inherits the advantages of the charge-balanced property of the HF DBS signal as well as the desynchronizing impact of the delayed feedback stimulation. Furthermore, we presented a detailed investigation of the pulsatile LDF and NDF when the stimulation pulses are equipped with an interphase gap [Fig. 3B]. The effects of the stimulation are illustrated when administered to a physiology-based model of interacting populations of excitatory STN and inhibitory GPe neurons suggested to model parkinsonian dynamics^62^.

We showed that both pulsatile linear and nonlinear delayed feedback stimulation can robustly and effectively desynchronize the stimulated neuronal population and revealed the desynchronization regions in the parameter plane of the stimulation delay *τ* and stimulation intensity *K*. Intriguingly, an interphase gap of finite width incorporated into the stimulation pulses can significantly improve the desynchronizing effects of the pulsatile delayed feedback stimulations as compared to the case without gap. The interphase gap leads to a better suppression of synchronization for the same values of the stimulation parameters. This effect is enhanced for longer gap, where the stimulation can induce stronger desynchronization as compared to shorter gaps. The amount of the administered stimulation required for full-blown desynchronization is much smaller for longer gaps. The desynchronizing action of the pulsatile delayed feedback stimulation with pulses with zero interphase gaps is somewhat limited as the parameter of the stimulation intensity increases [Figs 8 and 9]. On the other hand, using the pulses with interphase gap of finite width allows to achieve indeed strong desynchronization, where the order parameter can reliably be reduced as the stimulation intensity increases [Figs 8 and 9]. We verified the robustness of the reported results for the case of slowly changing stimulation delay *τ* modeling the variation of the LFP oscillation frequency, see Supplementary Section S4, as well as for the case of weakly coupled neurons exhibiting weak and intermittent synchronization, where the order parameter and LFP oscillations show more physiological variability in amplitude and phase, see Supplementary Section S5. We found that for an appropriate selection of the stimulation parameters causing a pronounced desynchronization of initially strongly synchronized neurons, the stimulation by pulsatile LDF and NDF preserves desynchronization when the neuronal population runs into a regime of weak or intermittent synchronization with a moderate variation of the firing frequency, e.g., caused by intrinsic variations of system parameters.

The smooth (i.e. non-pulsatile) LDF and NDF techniques have been tested for many different models and stimulation setups, demonstrating a pronounced desynchronizing effect^46, 47, 50, 54, 56, 76, 78, 81^. The structure of the parameter space of LDF was also experimentally confirmed for arrays of coupled electrochemical oscillators^82^. The desynchronizing impact of smooth NDF was also confirmed experimentally in the context of the suppression of alpha rhythm in the visual cortex by visual stimulation in healthy subjects^83^. The present study shows that pulsatile delayed feedback stimulation with an appropriate interphase gap can robustly induce a pronounced desynchronization. This provides a starting point for further translational studies counteracting abnormal neuronal synchronization in the framework of closed-loop DBS. Apart from its translational potential this study illustrates how important it is to vary basic stimulation parameters and features, such as the interphase gap, in order to better stimulation approaches. Another example that illustrates how important it is to scrutinize standard stimulation parameters was provided by Reich *et al*.^84^ who showed that short pulse width widens the therapeutic window of HF DBS delivered through the STN.

### Limitations

There are a couple of limitations we have to take into account from the theoretical and translational standpoint. Appropriate consideration of these limitations may provide guidance for future theoretical as well as translational studies. The considered model is a well-established model for parkinsonian dynamics and was used in a number of studies^62, 63, 73, 85^. From a dynamical standpoint, it is already complex to start with. However, with respect to neuroanatomy and neurophysiology it has limitations. For instance, it does not take into account the cortical involvement in the abnormal synchronization process as e.g. revealed by Oswal *et al*.^86^. The investigated methods of pulsatile delayed feedback stimulation can also be tested on more sophisticated and complicated models including further brain regions. However, the more complex a model is, the more difficult it gets to thoroughly study its dynamics and come up with reasonably reliable and general predictions. This is why we choose a different approach and introduce a stimulation technique in simpler, even minimal models such as phase oscillator network, derive predictions, test the latter in models of increasing complexity (see e.g. refs 28, 30, 33, 37, 85) and finally contribute to pre-clinical and clinical tests. The goal of the stepwise, computational top-down approach is to come up with robust predictions, tested for different levels of model complexity and stimulus action. There is increasing experimental evidence that stimuli might act antidromically at the cortical level (see e.g. ref. 13, 87). Given the anatomical proximity of different stimulated structures, future studies should be devoted to investigate whether and, if so, for which parameter ranges the dynamic stimulation mechanisms described here are still valid for antidromic, direct somatic, excitatory synaptic, inhibitory synaptic stimulation and mixtures thereof (compare ref. 37). By the same token it will be important to computationally test the stimulation technique proposed in this paper in network models with STDP^31, 32^. Computationally, it was shown that CR stimulation^28, 29^ can induce an anti-kindling, i.e. a long-lasting desynchronization and down-regulation of synaptic weights^30, 33^. Long-lasting desynchronization is of great clinical relevance^40–42^. However, since the dynamics of networks with STDP is considerably more complex (see e.g. ref. 88), the effects of pulsatile LDF and NDF still remain to be tested in neural networks with STDP. In the context of possible cortical stimulation effects, it is interesting that a similar network model composed of two neuronal populations of excitatory and inhibitory neurons was suggested for modeling epileptic seizures^89^, where the demand-controlled character of the pulsatile delayed feedback stimulation can be of special relevance because of the episodic nature of seizures.

Our approach requires that an oscillatory biomarker, a quantity sufficiently representing both abnormal neuronal synchrony and the patient’s state and conditions, exists. For closed-loop DBS, different types of feedback signals and observables have been used to trigger stimulation^64–67^ or to adapt stimulation intensity^72^. However, several open issues remain^80, 90^, and it is questionable whether beta LFP power alone might provide such a biomarker^91^, whether it is found consistently in patients^90, 92^, especially of different phenotype^93^, and sufficiently well represents clinical scores^94, 95^ or whether relations and/or interactions of different rhythms have to be taken into account^80, 95^. Furthermore, our approach requires that the oscillatory biomarker can be recorded. However, because of the large stimulation-induced artifact^90^, it could be difficult to perform the pulsatile LDF and NDF with the same stimulation and recording electrode. To remove stimulation-induced artifacts, a special filtering has to be applied^69^, or the stimulation and recording can be arranged by adjacent contacts in a specific bipolar configuration^66^. Accordingly, to translate our theoretical findings into the clinical arena we can further extend our approach to separate stimulation-registration setups as used in the context of desynchronizing proportional-integro-differential feedback^51^ or desynchronizing mixed NDF^56^ or to an act-and-wait protocol^96^.

However, the approach presented in this paper might ultimately help to study the functional role of possible oscillatory biomarker candidates and help reduce side effects by inducing long-lasting, sustained therapeutic effects, as shown in pre-clinical studies^40, 41^ and a clinical proof of concept study^42^ on CR-DBS. However, if the pulsatile delayed feedback methods presented here will actually elicit sustained long-lasting effects, we might contemplate different closed-loop control modes. In fact, different types of stimulation will require different closed-loop control variants. For instance, for desynchronizing stimulation it might make sense to (re-)calibrate in a closed-loop manner. In contrast, the simple on/off-type of demand-controlled stimulation might not necessarily be an optimal candidate control mode. In fact, computationally it was shown that specifically designed open loop pausing patterns that are not related to the amount of neuronal synchrony may significantly potentiate the desynchronizing effect of even very weak desynchronizing stimulation^38^.

It would be interesting to compare the efficacy in desynchronizing pathological neuronal oscillations of the methods considered in this paper to other control methods, for instance, to those relying on event-based stimulation^57, 58, 65, 66^, where the stimuli are administered when, for example, LFP amplitude exceeds a certain threshold, or phase-locked stimulation^27, 97, 98^, where the stimuli are administered at a particular phase of the oscillation cycle. In a clinical study^97^ in 10 patients with essential tremor, stimuli administered at an optimal tremor phase caused a tremor reduction of 27%. Improvement in tremor severity by the standard HF DBS was on average 70%, supporting satisfactory DBS electrode placement. This approach might be further improved by taking into account the dependence on the tremor amplitude itself^27^.

In conclusion, we showed that pulsatile LDF and NDF can effectively and robustly desynchronize a neuronal network of STN-GPe model neurons. Introducing an interphase gap between cathodic and anodic phases of the biphasic charge-balanced electrical pulses can enhance the efficacy of the stimulation, where a stronger desynchronization can be achieved at weaker stimulation intensities. Our results may contribute to the further development of methods for the treatment of neurological diseases characterized by abnormal neuronal synchronization in the framework of closed-loop deep brain stimulation.

## Electronic supplementary material

Supplementary Information
