# Supplementary material for: Closed-loop deep brain stimulation by pulsatile delayed feedback with increased gap between pulse phases
Source: Sci Rep. 2017 Apr 21;7:1033. doi: 10.1038/s41598-017-01067-x (PMC5430852; doi:10.1038/s41598-017-01067-x)
Supplement: Supplementary file 1 — Supplementary Information [file 41598_2017_1067_MOESM1_ESM.pdf]

Closed-loop deep brain stimulation by pulsatile delayed feedback with increased gap between pulse phases

Oleksandr V. Popovych[1,*], Borys Lysyansky[1], and Peter A. Tass[1,2,3]

[1]Institute of Neuroscience and Medicine - Neuromodulation, Jülich Research Center, Jülich, Germany
[2]Department of Neurosurgery, Stanford University, Stanford, California, USA
[3]Department of Neuromodulation, University of Cologne, Cologne, Germany
[*]o.popovych@fz-juelich.de

Supplementary information

S1 Parameters of the considered model of STN-GPe network

The values of parameters of the neuronal model (1) - (3) are listed in Table S1. The model was originally introduced in paper[1], and the values of parameters mostly coincide with those from the papers[1–3] and are taken to demonstrate the synchronous bursting dynamics of the STN neurons, which is a hallmark of the pathological parkinsonian state[4,5].

S2 Pulsatile NDF and LDF with increased stimulation intensity

We illustrate in more detail how the order parameter R of STN neurons stimulated by pulsatile NDF behaves when parameter of the stimulation intensity K increases. As has also been reported for other models[6–8] the order parameter decays $\sim K^{\gamma}$, as illustrated in Fig. S1. For the considered model containing N=200 STN neurons, a direct fit of the curves in Fig. S1A gives $\gamma \approx -0.3$ for an intermediate range of K, see the straight dashed line in Fig. S1A. The situation can differ for large stimulation intensity, especially, for interphase gaps of a moderate width. We found that for such a range of parameters, the order parameter can decay somewhat faster with smaller exponent γ. For example, $\gamma \approx -0.41$ for the gap width $GW = 1$ ms, or $\gamma \approx -0.51$ for $GW = 2$ ms, as illustrated in Fig. S1B.

The same scaling also holds for the amplitude of the filtered LFP, as illustrated in Fig. S1C, which is used to construct the NDF stimulation signal S of the form (7). Since the LFP enters to the NDF stimulation signal in the power of 3, the amplitude of the stimulation signal S will increase (decrease) with increasing K if the exponent $\gamma > -1/3$ ($\gamma < -1/3$), whereas the order parameter R always decays [Fig. S1]. Therefore, as the order parameter decreases for increasing K, the amount of the administered stimulation $\langle |S| \rangle$ grows for an intermediate range of R, whereas both R and $\langle |S| \rangle$ may decay for smaller R and for a moderate width of the interphase gap, as illustrated in Fig. 9B of the paper.

For the pulsatile LDF stimulation the situation is somewhat similar, where the decay rate of the order parameter may slightly increase for large stimulation intensity and for interphase gaps of a moderate width, see Fig. S1D. This however may not suffice for a consistent decay of the amount of the administered stimulation as reported in Fig. 9A of the paper.

S3 High-frequency deep brain stimulation

We stimulate the considered model by a high-frequency (HF) pulse train of the considered charge-balanced pulses with constant amplitude, which corresponds to a constant modulating signal $S(t) = K$ in Fig. 3 and models the standard HF deep brain stimulation (DBS).

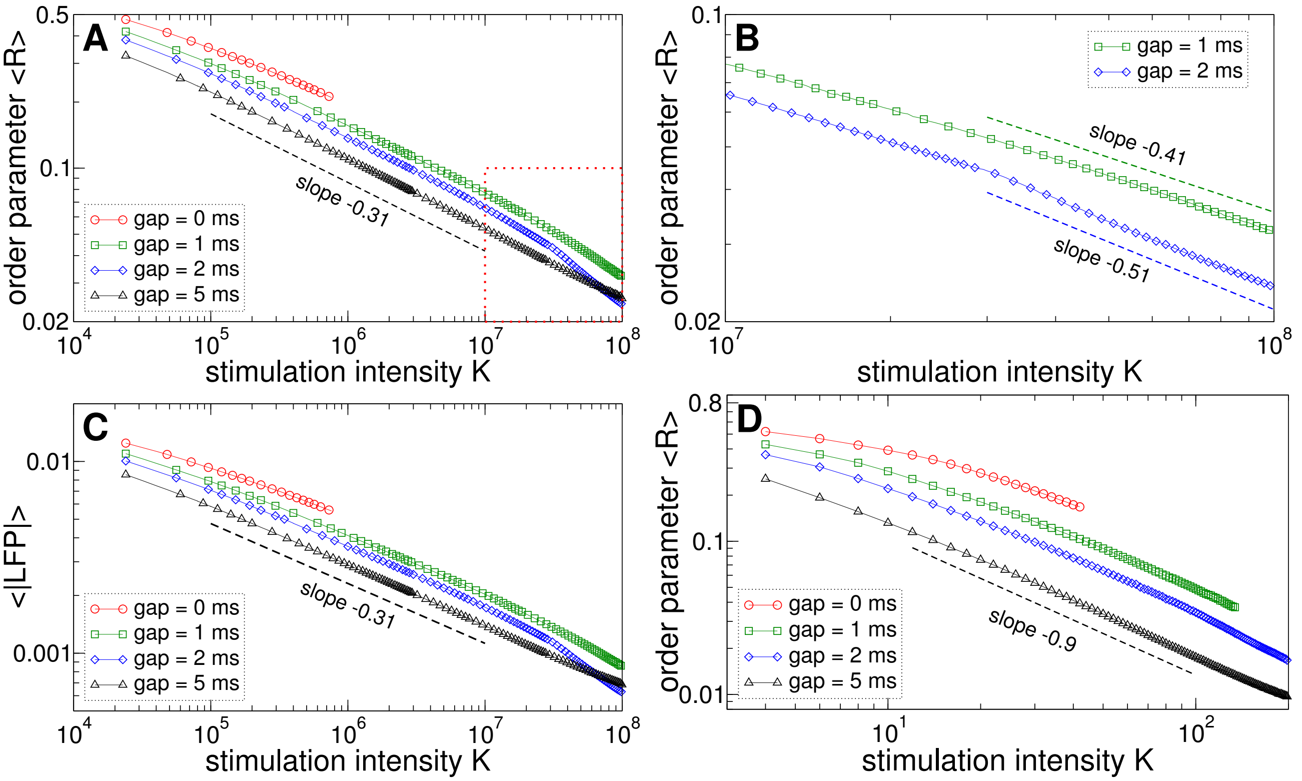

Figure S1. **Desynchronization of the neuronal ensemble (1) - (3) by pulsatile NDF and LDF for large stimulation intensity.** Time-averaged order parameter $\langle R \rangle$ of STN neurons is plotted in the log-log scale versus parameter K of the stimulation intensity for different widths of the interphase gap as indicated in the legends for (**A**), (**B**) pulsatile NDF and (**D**) pulsatile LDF. The part of plot (**A**) delineated by red dotted rectangle is enlarged in plot (**B**). (**C**) Time-averaged absolute value $\langle |LFP| \rangle$ of the filtered LFP corresponding to plot (**A**). The dashed lines have the slopes indicated in the plots and obtained by a direct fit of the corresponding curves. Stimulation delay (**A**) - (**C**) $\tau = 150$ ms and (**D**) $\tau = 70$ ms.

Parameter	STN	GPe	Units	Parameter	STN	GPe	Units
$g_{\rm L}$	2.25	0.1	nS/μm^2	θ_m	-30.0	-37.0	mV
$g_{\rm K}$	40	30	nS/μm^2	θ_h	-39.0	-58.0	mV
$g_{\rm Na}$	50	120	nS/μm^2	θ_n	-32.0	-50.0	mV
$g_{\rm T}$	0.5	0.5	nS/μm^2	θ_r	-67.0	-70.0	mV
$g_{\rm Ca}$	0.5	0.15	nS/μm^2	θ_a	-63.0	-57.0	mV
$g_{\rm AHP}$	9.0	30	nS/μm^2	θ_b	0.4	–	
$v_{\rm L}$	-60.0	-55.0	mV	θ_s	-39.0	-35.0	mV
$v_{\rm K}$	-80.0	-80.0	mV	θ_h^τ	-57.0	-40.0	mV
$v_{\rm Na}$	55.0	55.0	mV	θ_n^τ	-80.0	-40.0	mV
$v_{\rm Ca}$	140.0	120.0	mV	θ_r^τ	68.0	–	mV
τ_h^1	500.0	0.27	ms	θ_g^H	-39.0	-57.0	mV
τ_n^1	100.0	0.27	ms	θ_g	30.0	20.0	mV
τ_r^1	17.5	–	ms	σ_m	15.0	10.0	mV
τ_h^0	1.0	0.05	ms	σ_h	-3.1	-12.0	mV
τ_n^0	1.0	0.05	ms	σ_n	8.0	14.0	mV
τ_r^0	40.0	–	ms	σ_r	-2.0	-2.0	mV
ϕ_h	5	0.1		σ_a	7.8	2.0	mV
ϕ_n	5	0.3		σ_b	-0.1	–	
ϕ_r	2	1.0		σ_s	8.0	2.0	mV
k_1	15.0	30.0		σ_h^τ	-3.0	-12.0	mV
$k_{\rm Ca}$	22.5	3.0		σ_n^τ	-26.0	-12.0	mV
σ_r^τ	-2.2	–	mV	σ_g^H	8.0	2.0	mV
α	5.0	2.0	ms^{-1}	β	1.0	0.045	ms^{-1}
$I_{{\rm app},j}$	$\mathcal{N}(10, 0.015^2)$	-4.24	pA/μm^2	τ_r	–	30	ms
C_m	1.0	1.0	pF/μm^2	ε_j	$5\cdot 10^{-5}$	$\mathcal{N}(0.0055, [2\cdot 10^{-5}]^2)$	ms^{-1}

Table S1. Parameter set of the considered model of STN-GPe network (1)-(3).

Examples of the time courses of the order parameter $R(t)$ of the STN neurons stimulated by the HF DBS are illustrated in Fig. S2A for fixed stimulation intensity $K = 2.1$ and width of the interphase gap $GW = 0$ ms (red curve), 1 ms (green curve) and 5 ms (black curve). After the onset of the stimulation at $t = 20$ s with the considered stimulation intensity, the synchronized collective dynamics of the STN neurons is perturbed, and the order parameter fluctuates either at still relatively large values for zero gap or decays for non-zero gaps and fluctuates at smaller values than that of the initial, pre-stimulation synchronized regime. In the latter cases, this indicates a stimulation-induced desynchronization. Introducing the interphase gap of a finite width can thus improve the desynchronizing impact of HF DBS.

The time-averaged order parameter $\langle R \rangle$ of the STN neurons stimulated by HF DBS is depicted versus stimulation intensity K in Fig. S2B for different widths of the interphase gap. Strong enough HF DBS can suppress synchronization to approximately the same extent as given by the values of the order parameter for zero and non-zero gaps. For stimulation at intermediate strength, however, introducing an interphase gap may improve the desynchronizing impact of HF DBS, as demonstrated by the values of the order parameter for zero gap [Fig. S2B, red circles] with respect to those for gap width $GW = 1$ ms [Fig. S2B, green squares]. Further increase of the interphase gap may not necessarily lead to stronger desynchronization, albeit it is still more favorable than zero gap stimulation, as illustrated by the values of the order parameter for $GW = 5$ ms in Fig. S2 (black curves).

In summary, in the considered model strong HF DBS can suppress the abnormal neuronal synchronization. Introducing an interphase gap in the stimulation biphasic charge-balanced pulses may improve the desynchronizing effect for intermediately strong stimulation. This may potentially allow to reduce the stimulation intensity of HF DBS without a significant worsening of its therapeutic effects. The extent of the stimulation-induced desynchronization may, however, strongly fluctuate as reflected by

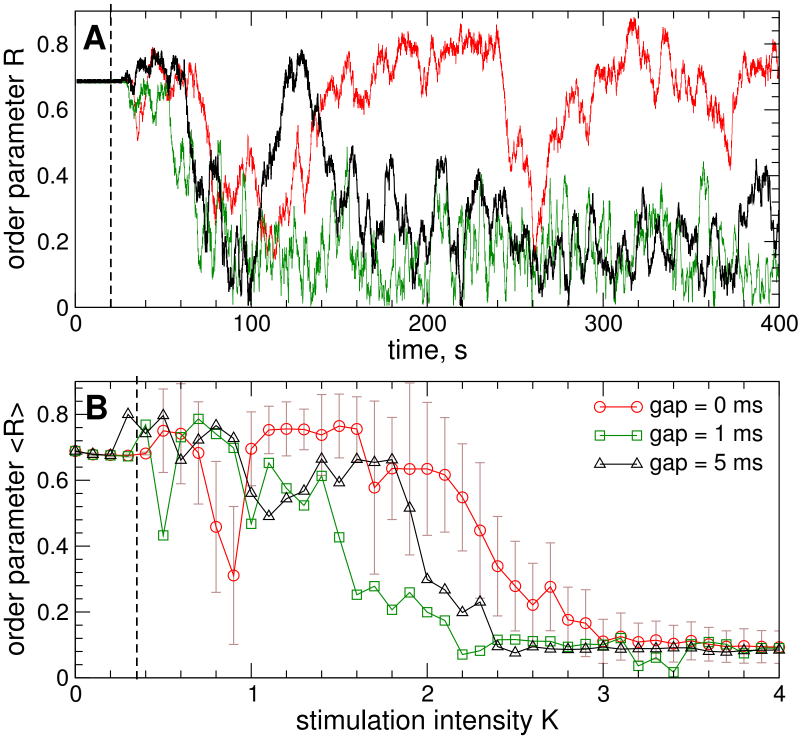

Figure S2. Suppression of synchronization in the neuronal ensemble (1) - (3) by HF DBS. (**A**) Time courses of the order parameter R of STN neurons for different widths of the interphase gap $GW = 0$ ms (red curve), 1 ms (green curve) and 5 ms (black curve). The stimulation starts at $t = 20$ s as indicated by the vertical dashed line, where the parameter of the stimulation intensity K linearly increases and reaches its maximal value $K = 2.1$ at $t = 70$ s. (**B**) Time-averaged order parameter $\langle R \rangle$ versus stimulation intensity K for different widths of the interphase gap as indicated in the legend. For zero gap the standard deviation of the order parameter fluctuations, see plot (**A**), is indicated by error bars. The vertical dashed line bounds the range of the stimulation intensity $K \in [0, 0.35]$ used to compare the efficacy of HF DBS and feedback methods in Fig. 9 of the paper.

the values of the order parameter [Fig. S2], and the improvement is less consistent if the width of the interphase gap further increases, as compared to the results reported for the pulsatile delayed feedback stimulations. It is important to note here that the amount of the administered stimulation utilized by HF DBS to desynchronize the stimulated neurons and calculated in this case as $\langle |S| \rangle = K$ is much larger than that of the pulsatile delayed feedback methods. In the latter cases the STN neurons get strongly desynchronized for $\langle |S| \rangle < 0.35$, see Fig. 9 of the paper, whereas HF DBS either does not change the extent of neuronal synchronization or can even enhance it for the same range of $\langle |S| \rangle = K$, see the range of $K \in [0, 0.35]$ bounded by the vertical dashed line in Fig. S2B.

S4 Slowly varying parameters

To further verify the robustness of the considered stimulation methods with respect to parameter variation, we simulate the considered model under continuous stimulation by pulsatile LDF or NDF and slowly vary the delay parameter τ. Since the optimal stimulation delay for desynchronization relates to the oscillation period of the mean field[6–10], such varying τ can model a slow variation of the firing frequency of the stimulated neurons. In our simulations, for example, the stimulation delay changed by 2 ms every 100 s such that the initial conditions of the model for the next value of τ were the last state of the system for the previous value of τ. The results of such a continuation by parameter τ are illustrated in Fig. S3, where the time-averaged order parameter $\langle R \rangle$ of the STN neurons is depicted versus the stimulation delay τ for pulsatile LDF [Fig. S3A, B] and NDF [Fig. S3C, D]. The variation of τ does not cause any problem with respect to the stimulation-induced desynchronization. Indeed, the pulsatile LDF stimulation with slowly varying delay [Fig. S3A, B, red circles and blue squares] demonstrates the same desynchronizing effects as for the case where the stimulation is administered to an initially synchronized population

[Fig. S3A, B, black solid curves]: The size and location of desynchronization regions are well preserved. For the pulsatile NDF stimulation the situation can even be improved, where the variation of parameters can significantly extend the desynchronization regions [Fig. S3C, D, red circles and blue squares]. With such an approach of slowly varying stimulation parameters, the NDF stimulation can desynchronize the stimulated neurons for practically all values of the stimulation delay as has also been reported for other models[6–8]. For the model studied in the present paper, the considered slow variation of the delay parameter allows for the NDF stimulation to explore other coexisting stable desynchronized regimes to which the stimulated neuronal population can be shifted by the pulsatile NDF, in such a way extending the desynchronization parameter regions.

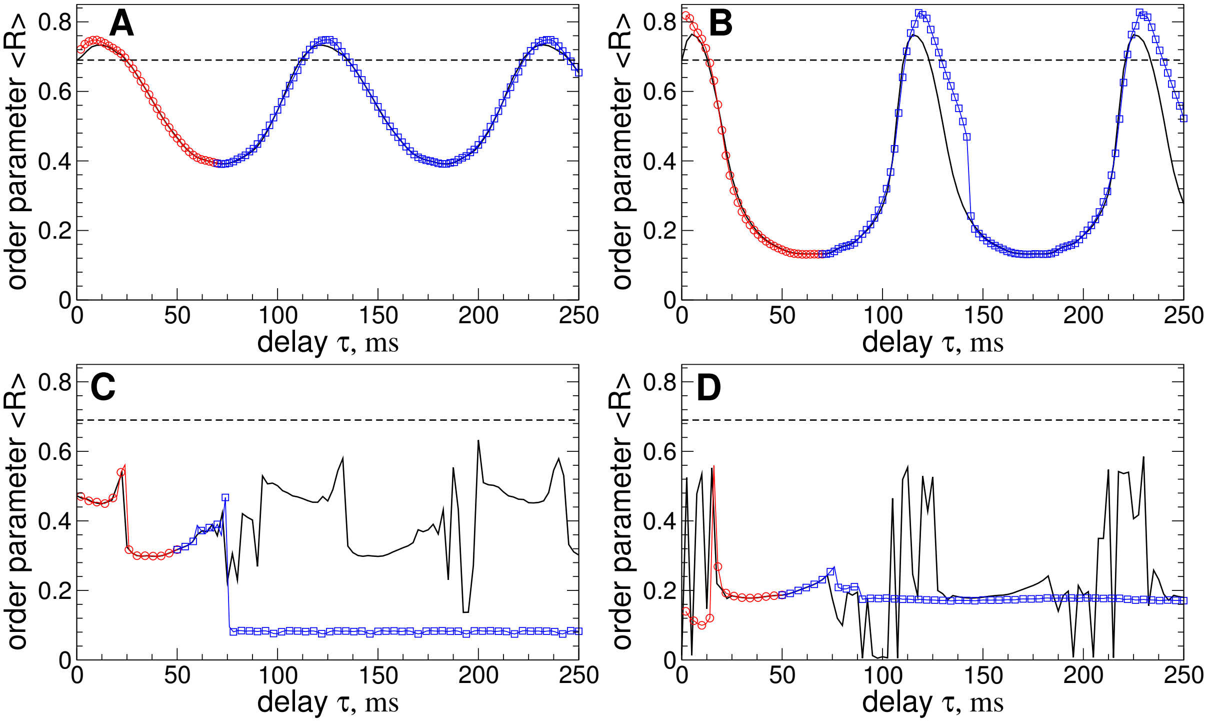

Figure S3. Synchronization control in the neuronal ensemble (1) - (3) by the pulsatile LDF and NDF stimulations for slowly varying stimulation delay τ. The time-averaged order parameter $\langle R \rangle$ of the stimulated STN neurons is plotted versus τ for (**A**), (**B**) pulsatile LDF and (**C**), (**D**) pulsatile NDF stimulation for the width of the interphase pulse gap (**A**), (**C**) $GW = 0$ ms and (**B**), (**D**) $GW = 5$ ms. The black solid curves depict the stimulation-induced values of $\langle R \rangle$ from Figs. 6 and 7, where, for each value of τ, the stimulation is administered to initially synchronized neurons. The red circles and blue squares depict the values of $\langle R \rangle$ obtained by continuation by parameter τ when it decreases and increases, respectively, starting from (**A**), (**B**) $\tau = 70$ ms and (**C**), (**D**) $\tau = 50$ ms. The horizontal dashed lines indicate the amount of synchronization in the stimulation-free STN. Parameter of the stimulation intensity (**A**), (**B**) $K = 10$ and (**C**), (**D**) $K = 2 \cdot 10^5$.

S5 Weakly and intermittently synchronized neurons

We also consider the case of weak coupling $g_{G \to S} = 1.28$ nS/μm^2 where the STN neurons exhibit a weak and intermittent synchronization, and the order parameter fluctuates around small values as illustrated in Fig. S4A. The local field potential (LFP) oscillates with the mean period $T \approx 100$ ms and with small and varying amplitude as illustrated in Fig. S4B. We consider such a regime of weak and intermittent synchronization as an initial state for stimulation by pulsatile delayed feedback.

The effect of the stimulation by pulsatile LDF and NDF is illustrated in Figs. S4C and S4D, respectively, for two interphase gaps $GW = 0$ ms and 5 ms as indicated in the legend. Since neurons were initially only weakly synchronized, the desynchronizing stimulation only slightly changes the extent of synchronization, where the order parameter of the stimulated neurons still fluctuates at similarly small values as without stimulation. We observe that introducing an interphase gap can improve the desynchronizing effect of the stimulation also in the case of weakly and intermittently synchronized neurons. For

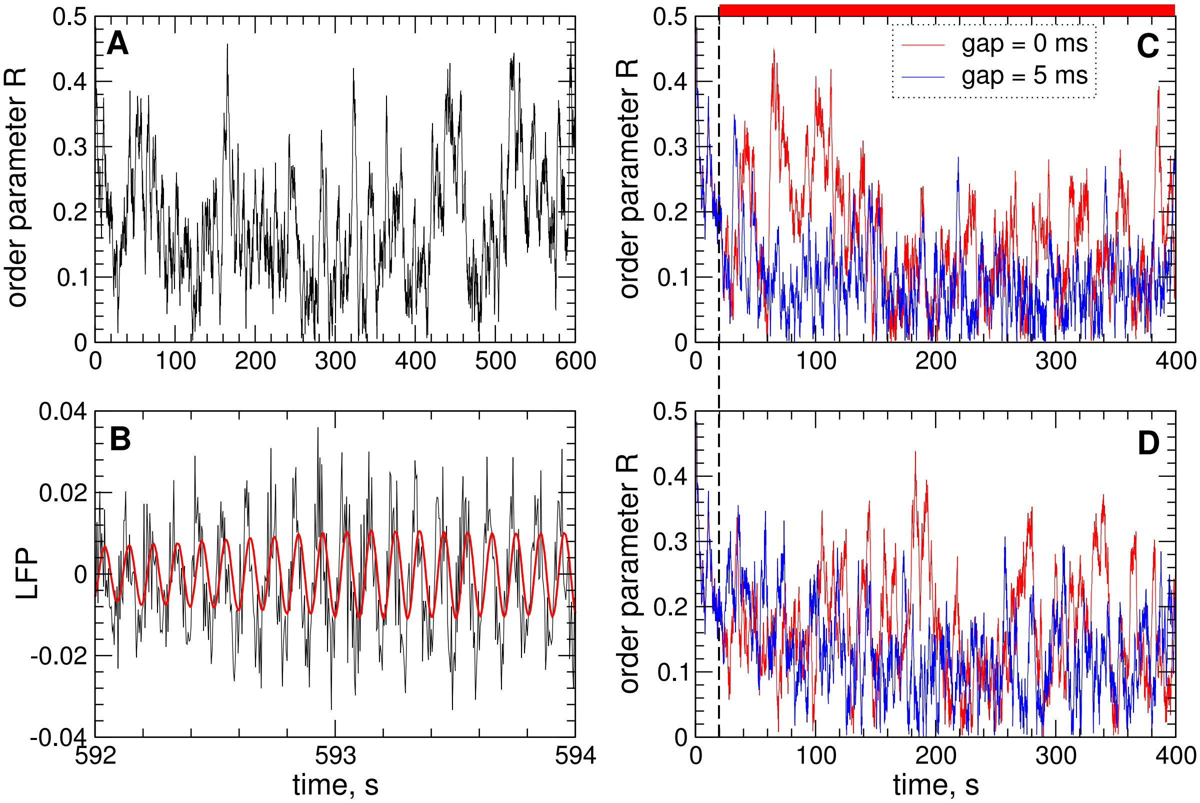

Figure S4. Stimulation of weakly synchronized neuronal ensemble (1) - (3) by pulsatile LDF and NDF stimulation. (**A**) Time course of the order parameter R and (**B**) the corresponding raw LFP (black curve) and filtered LFP (red curve) of the STN neurons for the stimulation intensity $K = 0$ (stimulation-free case). (**C**), (**D**) Time courses of the order parameter of the STN neurons stimulated by (**C**) pulsatile LDF and (**D**) pulsatile NDF with the interphase gap of the width indicated in the legend. The stimulation epochs started at $t = 20$ s as indicated by vertical dashed line and the red bar on the top of the plot (**C**). The neurons are weakly coupled with $g_{G\to S} = 1.28$ nS/μm^2. Parameters $\tau = 50$ ms, and $K = 15$ in plot (**C**) and $K = 10^6$ in plot (**D**).

example, for the stimulation parameters of the pulsatile LDF as in Fig. S4, the time-averaged order parameter $\langle R\rangle \approx 0.14$ for $GW = 0$ ms [Fig. S4C, red curve], 0.13 for $GW = 1$ ms, 0.11 for $GW = 2$ ms, and 0.1 for $GW = 5$ ms [Fig. S4C, blue curve]. For the pulsatile NDF stimulation, $\langle R\rangle \approx 0.16$ for $GW = 0$ ms [Fig. S4D, red curve], 0.14 for $GW = 1$ ms, 0.13 for $GW = 2$ ms, and 0.11 for $GW = 5$ ms [Fig. S4D, blue curve]. In the stimulation-free case the order parameter $\langle R\rangle \approx 0.19$ [Fig. S4A]. The observed differences of the order parameter are statistically significant as revealed by the Wilcoxon rank sum test applied to all pairs of the gap widths for LDF and NDF including the stimulation-free case. The maximal p-value for all pairs was obtained to be smaller than 0.001 even for time series of the order parameter downsampled to the sampling rate of 5 Hz.

To illustrate the desynchronizing impact of the pulsatile LDF and NDF stimulations administered to weakly coupled and weakly synchronized STN neurons, we scan the parameter space (τ, K) of the stimulation delay τ and the stimulation intensity K and depict the time-averaged order parameter $\langle R\rangle$ of the stimulated neurons in color in Figs. S5A and S5B for the pulsatile LDF and NDF stimulations, respectively. We found that the desynchronizing effect of both pulsatile LDF and NDF stimulations is robust with respect to the variation of the extent of synchronization and frequency in the neuronal population. Indeed, for the same stimulation parameters as for the initially strongly coupled and synchronized neurons, the pulsatile LDF stimulation does not cause any enhancement of synchronization in the ensembles of initially weakly coupled and weakly synchronized neurons, see Fig. S5A and compare with Fig. 6B in the paper. Also for the pulsatile NDF stimulation, the desynchronization regions obtained for initially strongly coupled and strongly synchronized neurons shown in Fig. 7B in the paper overlap well with the desynchronization regions for initially weakly coupled and weakly synchronized neurons, see Fig. S5B (blue domain).

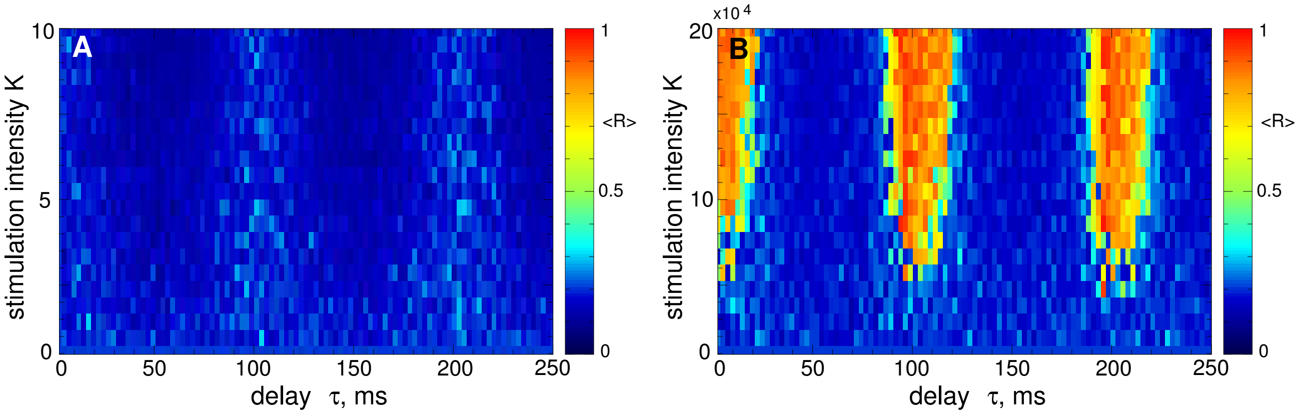

Figure S5. Impact of the pulsatile LDF and NDF stimulations on the weakly coupled and weakly synchronized neuronal ensemble (1) - (3). The time-averaged order parameter $\langle R(t) \rangle$ of the stimulated STN neurons is depicted in color ranging from 0 (blue) to 1 (red) versus the feedback delay τ and the stimulation intensity K for a weakly coupled regime with $g_{G \to S} = 1.28$ nS/μm^2 for (**A**) pulsatile LDF and (**B**) pulsatile NDF. The width of the interphase gap $GW = 5$ ms.

Therefore, for an appropriate selection of the stimulation parameters causing a pronounced desynchronization of initially strongly synchronized neurons, the stimulation by pulsatile LDF and NDF preserves desynchronization when, e.g., due to variations of system parameters the neuronal population runs into a regime of weak or intermittent synchronization with a moderate variation of the firing frequency.

References

1. Terman, D., Rubin, J. E., Yew, A. C. & Wilson, C. J. Activity patterns in a model for the subthalamopallidal network of the basal ganglia. *J. Neurosci.* **22**, 2963–2976 (2002).
2. Rubin, J. E. & Terman, D. High frequency stimulation of the subthalamic nucleus eliminates pathological thalamic rhythmicity in a computational model. *J. Comput. Neurosci.* **16**, 211–235 (2004).
3. Park, C., Worth, R. M. & Rubchinsky, L. L. Neural dynamics in parkinsonian brain: The boundary between synchronized and nonsynchronized dynamics. *Phys. Rev. E* **83**, 042901 (2011).
4. Hammond, C., Bergman, H. & Brown, P. Pathological synchronization in Parkinson's disease: networks, models and treatments. *Trends Neurosci.* **30**, 357–364 (2007).
5. Benabid, A. L., Chabardes, S., Mitrofanis, J. & Pollak, P. Deep brain stimulation of the subthalamic nucleus for the treatment of Parkinson's diseaseegge. *Lancet Neurol.* **8**, 67–81 (2009).
6. Popovych, O. V., Hauptmann, C. & Tass, P. A. Effective desynchronization by nonlinear delayed feedback. *Phys. Rev. Lett.* **94**, 164102 (2005).
7. Popovych, O. V., Hauptmann, C. & Tass, P. A. Control of neuronal synchrony by nonlinear delayed feedback. *Biol. Cybern.* **95**, 69–85 (2006).
8. Popovych, O. V. & Tass, P. A. Synchronization control of interacting oscillatory ensembles by mixed nonlinear delayed feedback. *Phys. Rev. E* **82**, 026204 (2010).
9. Rosenblum, M. G. & Pikovsky, A. S. Controlling synchronization in an ensemble of globally coupled oscillators. *Phys. Rev. Lett.* **92**, 114102 (2004).
10. Rosenblum, M. G. & Pikovsky, A. S. Delayed feedback control of collective synchrony: An approach to suppression of pathological brain rhythms. *Phys. Rev. E* **70**, 041904 (2004).
